# Supplementary material for: A Decellularized Human Limbal Scaffold for Limbal Stem Cell Niche Reconstruction
Source: Int J Mol Sci. 2021 Sep 17;22(18):10067. doi: 10.3390/ijms221810067 (PMC8471675; doi:10.3390/ijms221810067)
Supplement: Supplementary file 1 [file ijms-22-10067-s001.zip › ijms-1356480-supplementary.pdf]

A decellularized human limbal scaffold for limbal stem cell niche reconstruction

Naresh Polisetti[1], Benjamin Roschinski[1], Ursula Schlötzer-Schrehardt[2], Philip Maier[1], Günther Schlunck[1], Thomas Reinhard[1]

Supplementary Figure S1: Uncropped version of Western Blot shown in Figure 1

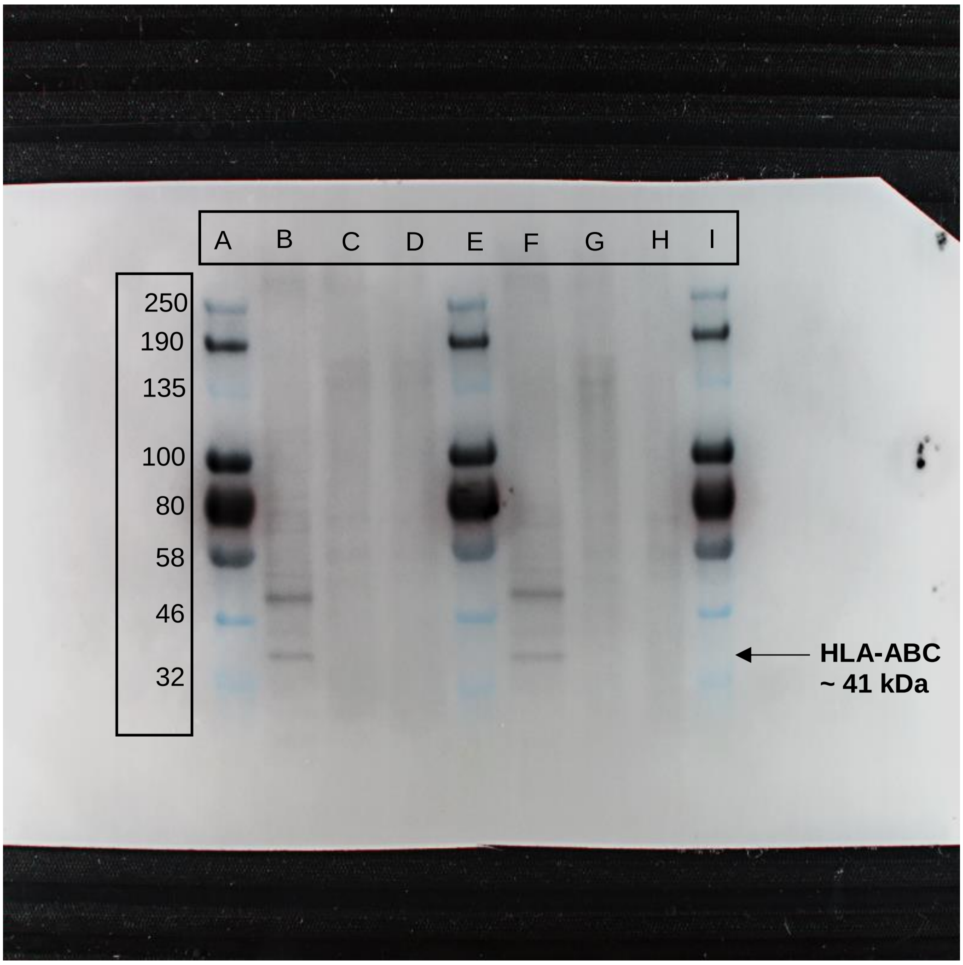

A, E, I – Ladder

B,F – Normal human limbus (NHL)

C, G – Decellularized human limbus (DHL) in 0% dextran

D, H – DHL in 4% dextran

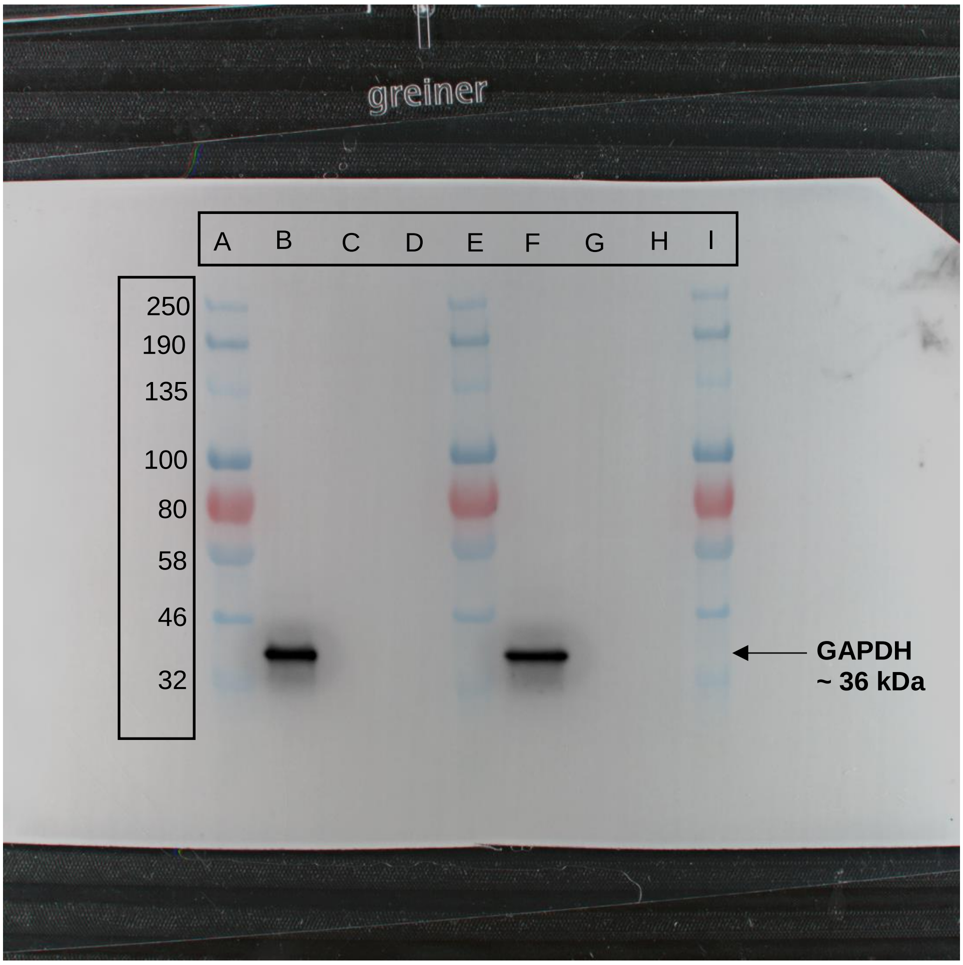

A, E, I – Ladder

B,F – Normal human limbus (NHL)

C, G – Decellularized human limbus (DHL) in 0% dextran

D, H – DHL in 4% dextran

Supplementary Figure S2: Ex vivo transplantation of DHL tissue. Abbreviations: NHL- normal human limbus; DHL – decellularized human limbus

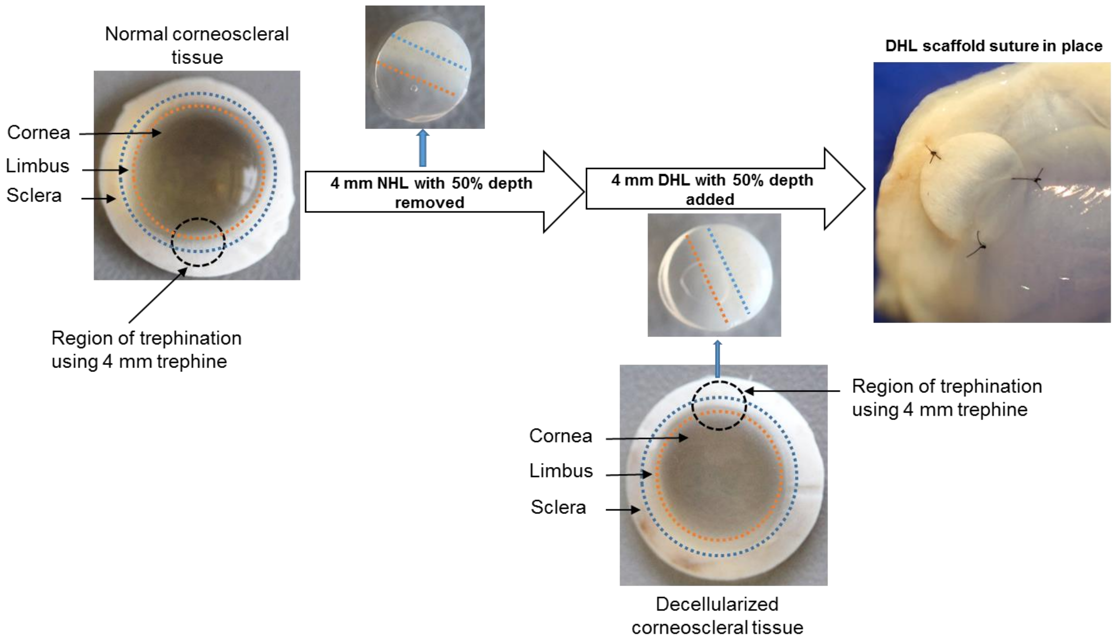

Supplementary Figure S3: Preparation of normal human limbus (NHL) and decellularized human limbal (DHL) scaffolds.

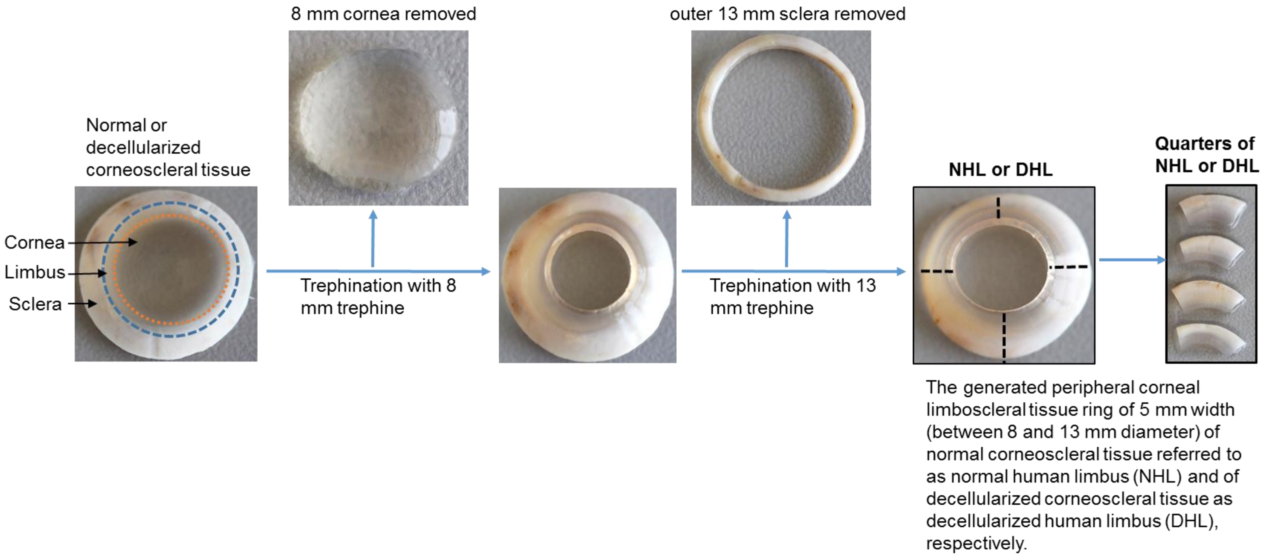

Supplementary Table S1. List of antibodies used

Antibody (clone), Host species	Antibody dilution	Application	Antibody source
Agrin, rabbit	1:2000	Immunohistochemistry	R. Timpl / T. Sasaki
Cadherin-E (24E10), rabbit	1:200	Immunohistochemistry	Cell signaling
Collagen III, rabbit	1:100	Immunohistochemistry	Abcam
Collagen IV (2F11), mouse	1:100	Immunohistochemistry	Southern Biotech
Collagen XVIII, rabbit	1:2000	Immunohistochemistry	R. Timpl / T. Sasaki
Cytokeratin pan (PCK-26), mouse	1:500	Immunohistochemistry	Abcam
Cytokeratin 3 (AE5), mouse	1:100	Immunohistochemistry	Abcam
Cytokeratin 15 (LHK15), mouse	1:500	Immunohistochemistry	Abcam
Cytokeratin 15 (EPR1614Y), rabbit	1:500	Immunohistochemistry	Abcam
HMB-45 (HMB45), mouse	0.5 µg/ml	Immunocytochemistry	Abcam
HLA-ABC (W6/32)	1:100	Immunohistochemistry/Western blot	Invitrogen
Fibronectin (IST-4), mouse	1:100	Immunohistochemistry	Sigma-Aldrich
JAM-C (CRAM-19H36), rat	1:100	Immunohistochemistry	Serotec (Biorad)
Ki-67 (EPR3610), rabbit	1:500	Immunohistochemistry	Abcam
Laminin α3, rabbit	1:1000	Immunohistochemistry	R. Timpl / T. Sasaki
Laminin α5 (504), rabbit	1:1000	Immunohistochemistry	L. Sorokin, Münster
Laminin β2 (409), rabbit	1:1000	Immunohistochemistry	L. Sorokin, Münster
Laminin β3, rabbit	1:1000	Immunohistochemistry	R. Timpl / T. Sasaki
Laminin γ2, rabbit	1:1000	Immunohistochemistry	R. Timpl / T. Sasaki
Melan A, (EPR20380), rabbit	1:1000	Immunohisto/cytochemistry	Abcam
p63 (Y289), rabbit	1:200	Immunohistochemistry	Abcam
Tenascin C, rabbit	1:200	Immunohistochemistry	Millipore
Vimentin, (D21H3), rabbit	1:500	Immunohistochemistry	Cell Signaling
Vitronectin (8E6), mouse	1:100	Immunohistochemistry	Millipore
